# Automated face recognition assists with low‐prevalence face identity mismatches but can bias users

**DOI:** 10.1111/bjop.12745

**Published:** 2024-11-15

**Authors:** Melina Mueller, Peter J. B. Hancock, Emily K. Cunningham, Roger J. Watt, Daniel Carragher, Anna K. Bobak

**Affiliations:** ^1^ Psychology, Faculty of Natural Sciences University of Stirling Stirling UK; ^2^ School of Psychology, Faculty of Health and Medical Sciences University of Adelaide Adelaide South Australia Australia

**Keywords:** attitudes towards AI, automated face recognition, decision making, deep neural networks, face matching, face recognition

## Abstract

We present three experiments to study the effects of giving information about the decision of an automated face recognition (AFR) system to participants attempting to decide whether two face images show the same person. We make three contributions designed to make our results applicable to real‐word use: participants are given the true response of a highly accurate AFR system; the face set reflects the mixed ethnicity of the city of London from where participants are drawn; and there are only 10% of mismatches. Participants were equally accurate when given the similarity score of the AFR system or just the binary decision but shifted their bias towards match and were over‐confident on difficult pairs when given only binary information. No participants achieved the 100% accuracy of the AFR system, and they had only weak insight about their own performance.

## BACKGROUND

Modern approaches to face identification involve both human decision‐makers and state‐of‐the‐art automated facial recognition system (AFR). While some user cases see the human operator provide oversight of decisions from the AFR, there are other circumstances in which the human operator uses the AFR as a decision‐making aid. There are many common tasks that require verifying a person's identity, such as buying alcohol, opening a bank account or travelling internationally. In these examples, verification typically involves a one‐to‐one face matching task, in which the current appearance of the person is compared to the photograph on their identification document (i.e. a passport or driver's licence), with the goal of determining whether they are an identity match (the same person) or a mismatch (two different people). Identity mismatches – when someone presents the photo ID of another person – are rare and often occur for nefarious purposes, including committing serious crimes (Franks & Smith, 2020; Syria Girls, 2015). However, identification errors, such as falsely declaring two different people to be the same person, can lead to significant miscarriages of justice including wrongful imprisonment, regardless of whether the decision is made by a human or AFR or both (Hill, 2020). Therefore, it is vital that these identifications are made as accurately as possible. However, both humans and AFR systems make errors when identifying faces. To test performance, studies in laboratory settings tend to employ designs where there is an equal number of matched and mismatched trials, mistakes are introduced by the researchers, and the sets of face images are of white people thus lacking ecological validity.

To address these gaps, we aimed to examine how people, aided by AFR, make face matching decisions under real‐world conditions. Specifically, when the so‐called mismatch prevalence is low (10%), the face set is constructed to reflect ethnicity of a big city (London) and where true performance of the AFR is disclosed to participants and without introducing ‘artificial’ mistakes.

The recent integration of deep convolutional neural networks (DCNN) into facial recognition technologies has led to significant advances in accuracy, such that these systems have now been incorporated into many face identification tasks. For example, the smart ‘e‐Gates’ found at border control in many airports incorporate AFR (Fysh & Bindemann, 2018a). The traveller places their passport image face down in a document scanner, and a camera in the gate captures their current appearance (MacLeod & McLindin, 2011). The AFR must locate a face in the submitted image, before creating a vector template that describes the face. By comparing the template of one face to that of another, the system returns a similarity score, usually the cosine of the angle between the vectors of the two compared faces. Based on a predetermined threshold, the system uses the similarity score to determine whether a match or a mismatch decision will be made. The accuracy of modern AFR algorithms often outperforms untrained individuals and experts alike, particularly on high‐quality imagery (Phillips et al., 2018; Phillips & O'Toole, 2014). However, these systems still make errors (Grother et al., 2019), some of which are obvious to humans (Hancock et al., 2020). As such, many workflows incorporate a human operator to oversee the decisions made by the AFR system (Howard et al., 2020). Though the intention is for humans to correct system errors before they are actioned, this task is surprisingly difficult (Carragher & Hancock, 2023).

Determining whether two photographs show the same person sounds trivially easy, and it is if the faces are familiar to the observer (Bruce et al., 2001; Noyes & Jenkins, 2019). It is a much different story, however, when the faces are unfamiliar to the observer (e.g. Megreya & Burton, 2006), as is the case in most applied settings. Average human performance on unfamiliar face matching tasks is often only 70–80% (e.g. Burton et al., 2010; White et al., 2015) and can be lower on more challenging tasks (Fysh & Bindemann, 2018b). Performance falls even further if there are variations to factors including viewpoint, image quality, colouration or ageing of the faces, among other manipulations (for review, see Fysh & Bindemann, 2017). This high error rate is particularly concerning when we consider that experience does not automatically improve performance; passport control officers (White et al., 2014), notaries (Papesh, 2018) and supermarket cashiers (Kemp et al., 1997) have all shown average face matching performance equivalent to that of untrained participants. Interestingly, Wirth and Carbon (2017) found German passport officers outperformed novices, in contrast to White et al. (2014), though their performance was negatively correlated with duration of employment – potentially suggesting that initial training might be responsible for their superior performance. There are, however, specialist groups that reliably show exceptional performance on matching tasks, including professional facial examiners (Phillips et al., 2018; White et al., 2015) and ‘super‐recognizers’ (Bobak et al., 2016; Robertson et al., 2016). Nonetheless, these findings suggest that many professionals responsible for performing face matching tasks are no better than the average novice.

So, humans and AFR both make errors when identifying faces – though modern AFR systems are often much better than the average human (Phillips et al., 2018). Yet, whether performing oversight, or using the AFR as a decision‐making aid, the human is often responsible for safeguarding against algorithm error. Even though both make errors, it is possible for the human‐algorithm team to experience a collaborative accuracy gain, whereby the performance of the team surpasses that of the human or the algorithm alone. Such a gain would be achieved if the human corrected AFR system's errors, while deferring to the system when they themselves have made an error (Bartlett & McCarley, 2017; Sorkin et al., 2001). But the few papers to investigate the performance of interacting human‐algorithm teams in one‐to‐one face matching tasks suggest that collaborative performance is far from ideal. Fysh and Bindemann (2018a) showed that humans were biased to follow the identification decision of the AFR; human accuracy was higher on trials that were answered correctly by the AFR and lower on trials that it was incorrect. Howard et al. (2020) also showed that human judgements of face similarity were higher when the AFR declared that face pairs showed the same person. This effect was later replicated by Barragan et al. (2022), who found this bias to be exaggerated when the faces were shown wearing lower face coverings, which also impair human face matching performance (Carragher & Hancock, 2020). Together, these studies suggest that humans are biased to follow the advice of the AFR, even when it is incorrect.

Although humans are biased to follow the decision of the AFR, they do not accept this advice uncritically. Carragher and Hancock (2023) had participants complete one‐to‐one face matching tasks when assisted by an AFR system that was based on the performance of a real DCNN. The authors also collected a baseline measure of human performance, to assess the benefit of teaming with the AFR system. Across five pre‐registered experiments, Carragher and Hancock (2023) found that most humans improved their face matching performance when assisted by an AFR with high accuracy (>90%). However, the performance of the average human‐AFR team was far worse than that of the same AFR alone. Not only did participants fail to detect errors from the AFR but they also overturned many of the system's correct decisions. This result occurred even though participants were informed of the exact accuracy of the AFR prior to the task. These findings are consistent with other examples of suboptimal human use of automated decision‐aids (Bartlett & McCarley, 2017; Boskemper et al., 2022) and point to a problem of *disuse* – underutilising a reliable decision‐aid (Parasuraman & Riley, 1997). Indeed, re‐analysis of these data revealed that the levels of AFR‐assisted face matching performance were consistent with the predictions of some of the least efficient models of collaborative decision‐making (Bartlett et al., 2023). But like face matching ability, further research has shown that there are significant individual differences in human‐algorithm teaming outcomes, with some participants able to achieve perfect performance when assisted by the AFR (Carragher et al., 2024).

While these studies have started to shed light on the way humans use AFR to assist in one‐to‐one face matching tasks, all have methodological features that limit generalizability to real‐world scenarios. First, only Carragher and Hancock (2023) based the performance of the AFR on a real DCNN system (see also Carragher et al., 2024). Other authors did not actually use an AFR at all, rather, they randomly allocated identity decisions (‘same’ or ‘different’) to the face pairs, while telling participants they came from an AFR (Barragan et al., 2022; Fysh & Bindemann, 2018a; Howard et al., 2020) or used the AFR to compare to humans (Phillips et al., 2018). Importantly, Carragher and Hancock (2023) showed that human accuracy is significantly correlated with the similarity values from the DCNN, such that humans and the AFR are likely to err on the same face pairs. Therefore, without using a real DCNN, experimental task performance is unlikely to generalize to applied settings, where AFR errors will not be random. Despite using a real DCNN, Carragher and Hancock (2023) were interested in testing whether humans could overturn errors, of which the real system made none. As such, they introduced algorithm errors by overturning trials that the DCNN resolved correctly but were closest to the algorithm's decision threshold. Therefore, none of the studies to date have investigated human‐algorithm teaming performance with the true output from a state‐of‐the‐art DCNN.

A second limitation of previous human‐algorithm teaming studies is one that is common to most face matching experiments. Standardized face matching tests often present participants with an equal number of match and mismatch trials (e.g. Burton et al., 2010). But in reality, identity mismatches occur far less frequently in applied settings (i.e. half of all travellers do not use somebody else's passport). Infrequent or rare targets result in poor target detection in visual search tasks (Wolfe et al., 2005) and are increasingly missed over time (e.g. Mackworth, 1948; McCarley & Yamani, 2021). Indeed, these low‐prevalence effects have also been found in face matching tasks, such that participants are more likely to miss identity mismatches when they occur infrequently (Papesh et al., 2018; Papesh & Goldinger, 2014). Papesh and Goldinger (2014) observed that when the prevalence of mismatches was low (10%), participants failed to detect nearly 50% of mismatches, compared to a miss rate of 25% when mismatches made up half of all trials. This low‐prevalence effect does not only occur for novices, having been observed for professional groups as well (Weatherford et al., 2021). To date, most human‐algorithm teaming experiments have presented participants with 50% mismatch trials (Barragan et al., 2022; Carragher & Hancock, 2023; Howard et al., 2020), meaning current findings might underestimate the true miss rate of false IDs. While Fysh and Bindemann (2018a, 2018b) had rare mismatch trials, their fictitious AFR system had an unrealistically low accuracy rate, which can discourage reliance on automated systems (Ross et al., 2008). Therefore, the current study will investigate how low‐prevalence effects influence dependence on realistic AFR system in face matching tasks.

Finally, nearly all human‐algorithm teaming studies to date have presented participants with AFR decisions that were binary (i.e. ‘same’ or ‘different’). But in real systems, these identification decisions are based on a ‘similarity value’ that the algorithm computes between the two images being compared. Appropriate use of automated decision‐aids requires the human operator to understand when to rely on the decision‐aid and when to use their own judgement (Parasuraman & Riley, 1997). The models of collaborative decision‐making that achieve optimal performance have the decision‐aid communicate direct evidence for the decision with the human decision‐maker, which in this case would be the similarity rating (Bahrami et al., 2010; Sorkin et al., 2001). Intuitively, this notion makes sense. A face pair that receives a similarity rating nearer to the system's threshold is more likely to be an error than a pair that is judged to be far from threshold. The human operator might reduce their reliance on the AFR when the similarity value is close to threshold and increase it when the value is far from threshold. Only Carragher and Hancock (2023) have examined the effect of providing a similarity value alongside a binary decision from the AFR (Experiment 1b), reporting no additional benefit of the supplementary similarity value compared to the binary decision alone. However, Carragher and Hancock's (2023) participants only made binary judgements. It is possible that any influence of the additional similarity value on decision‐making might only be evident when participants are asked to make a more fine‐grained, confidence‐style, judgement.

The overarching aim of the current study is to test the one‐to‐one face matching performance of human‐algorithm teams under conditions with greater real‐world validity. First, we have designed a face matching test that has a mismatch rate of only 10%, to examine the effect of low mismatch prevalence on AFR reliance. Second, we have based the performance of the AFR in the experiment on the true results of a real state‐of‐the‐art DCNN, without introducing additional errors. Third, we will test the effect of presenting participants with the similarity values from the AFR alongside a binary identification decision, while having participants make 6AFC responses that include both an identification decision (same, different) and an associated confidence (definitely, probably, guess). Finally, we have created our new face matching test to reflect the current ethnicity demographics of the greater city of London. Humans have previously been shown to have poorer face matching performance for faces of an ethnicity different to their own (e.g. Megreya et al., 2011), while some AFR systems can be biased against ethnicities that were not included in the DCNNs' training set (Cook et al., 2019; Phillips et al., 2011). Taken together, these methodological changes mean that our results will be highly generalizable to applied settings.

Although pre‐registered as separate, consecutive studies, the first two experiments are tightly linked and are presented here as Experiments 1a and 1b. Experiment 2 was designed to clarify some observations from Experiment 1. All three used identical participant recruitment, so we describe that first.

## GENERAL METHOD

### Transparency and ethics

All experiments, including their hypothesis, design and analysis, were pre‐registered on Open Science Framework before data collection. Pre‐registrations can be found here for Experiments 1a, 1b and 2. The study was approved by University of Stirling General Ethics Panel. Participants gave informed consent and were debriefed at the end of the study. They received £4.5 for completing the study, based on the standard rate of £9/h on Prolific and expected completion time of around 30 mins.

### Participants

Participants in all experiments were recruited using Prolific (an online recruitment platform where people can sign up to do studies; https://www.prolific.co/). They were required to be over the age of 18, live in London and have not participated in any of the other experiments or pilot tests. To make sure that participants would perform the study reliability, they were required to have taken part in at least five other studies on Prolific before and completed at least 90% of them. They also needed to take between 7 min and 1 h to complete the study for their data to be included. Since some of the faces used were of famous Polish personalities, participants were asked whether they were familiar with Polish media. If yes, their data were excluded. Furthermore, data of participants who started the study multiple times or did not finish it were also excluded. Failed attention checks (see below for more details) could also lead to the participant's exclusion.

We asked our participants to self‐describe their gender identity and ethnicity; the full breakdown is in supplementary analyses. While the modal response was White, less than half the participants described themselves thus.

#### Experiment 1a

Carragher and Hancock (2023) reported a very large effect size for using an AFR aid. For a conservative effect size of 0.4, with power of 0.8, G‐Power gave a sample size of 52. Allowing for 10% of exclusions, we recruited 57 from Prolific. Three were excluded for being familiar with famous Polish personalities. This left 54 participants (26 male, 28 female) average age 39, SD 11.8, who took an average of 22 min.

#### Experiment 1b

After exclusions, we had 56 participants (20 male, 35 female, 1 non‐binary) average age 40, SD 11.5, who took an average of 22 min.

### Design

A within‐participant design was used for each experiment. Participants were shown each face pair once without any additional information and once with the AFR system's input [infoType: without (unaided) or with AFR input] and had to rate the pair both times. Additionally, there are two types of trials in the task – matched trials (where two images show the same identity, known as *mated* in the AFR community) and mismatched trials (*non‐mated*, with two images showing two different identities) (faceType: match or mismatch).

### Face set

The face set was created for this task, consisting of 160 male face pairs, previously also used in Bate et al. (2018, 2019). It was aimed to be reflective of naturally occurring ethnicities. For this purpose, the 2021 census from London (https://apps.london.gov.uk/census‐2021‐reports/#/home/) was used to determine the prevalence of different ethnicities within the face set (54% White, 14% Black, 21% Asian, 11% Mixed/Other). The white faces were of Polish media personalities, while the other photos were downloaded from international model agencies websites. Only 16 of the face pairs (10%) were mismatches. All images were cropped to 300 × 420 pixels and are shown in colour. The face sets were piloted to check for their difficulty. Based on the response of 30 participants, the average accuracy for the face pairs ranged from 23% to 97% (*M* = 64%, *SD* = 16%).

### AFR system

A research AFR system from Imperial College, London, was used (https://insightface.ai/). When applied to the face set, it had an accuracy of 100%. The returned similarity scores ranged from −0.1 to 1, with a value over 0.3 indicating the faces are a match. For presentation to participants, we transformed the similarity scores so that the threshold was easier to understand 0.5. Values between 0.3 and 1 were transformed linearly to 0.5–1, while the range from −0.1 to 0.3 was transformed to 0–0.5. Unlike other studies, we did not introduce ‘mistakes’, but included the algorithm's real scores and informed participants that its accuracy on similar tests was over 99.9%.

### Attention checks

To make sure that participants pay attention, two face pairs of famous people were added on top of the 160 face pairs in the face set. One pair was matching, consisting of two photos of King Charles III (current King of the UK), and one was mismatching, showing Rishi Sunak (current UK prime minister) and Kunal Nayyar (British‐Indian actor). If participants got both pairs wrong, their data were excluded from the study.

### Procedure

#### Experiments 1a and 1b

The studies were completed online using Testable (an online software for stimulus presentation in experiments, https://www.testable.org/) and needed to be accessed from a computer. After collecting participant's demographic data (age, gender, ethnicity and familiarity with Polish media), they were instructed about the face matching task. Besides an explanation of what they would see on the following pages, they were told that around 10% of the faces will be mismatches and that the AFR system used for this study had shown an accuracy of over 99.9% on previous face pairs.

Participants were then shown the 162 face pairs in a random order. Each face pair was first shown without any additional information (text above the pair simply said ‘No computer response’) and participants had to rate whether the pair showed the same person on a 6‐point scale: 1, Definitely Different; 2, Think Different; 3, Guess Different; 4, Guess Same; 5, Think Same; and 6, Definitely Same. They were then shown the same pair again. This time, on top of the face pair, additional information from the AFR system was presented: AFR's similarity score in Experiment 1a and AFR's binary decision in Experiment 1b (match or mismatch). Participants were asked to rate on the same scale again, before being presented the next face pair. For participants in Experiment 1a, what the similarity score means, its range of values, and the threshold was explained at the start of the study.

Participants in Experiment 1b were asked additional questions after completing the face matching. First, to estimate their own unaided accuracy and that of the computer, as percent correct. Then, to answer 10 questions relating to their trust in the use of AI adapted from Ezzeddine et al. (2023), responding on a 5‐point scale from strongly disagree to strongly agree. This gave a trust score from 10 to 50. The questions are listed in Data S1 and in the pre‐registration.

## RESULTS

Participants' ratings were converted into binary decisions, by defining a rating from 1 to 3 as a mismatch (different) decision and a rating from 4 to 6 as a match (same) judgement. The binary scores were then used to calculate participants' overall accuracy and Hit and False‐Alarm rates. These were used to calculate their criterion and sensitivity d’ in accordance with Stanislaw and Todorov (1999). Hit or False‐Alarm rate values of 0 or 1 were adjusted, replacing 0 with 0.5/*n* and 1 with (*n* − 0.5)/*n*, where *n* is the number of matching/mismatch face pairs. Note that we define a hit as a correct match; some authors (e.g. Papesh et al., 2018) define a hit as a correct mismatch, which reverses the sign of criterion.

Where there are pre‐registered analyses that we have omitted from the main text, they are in supplementary analyses for completeness. Data and analysis files are available in the repository (bit.ly/3tQ3fC9).

**FIGURE 1:**
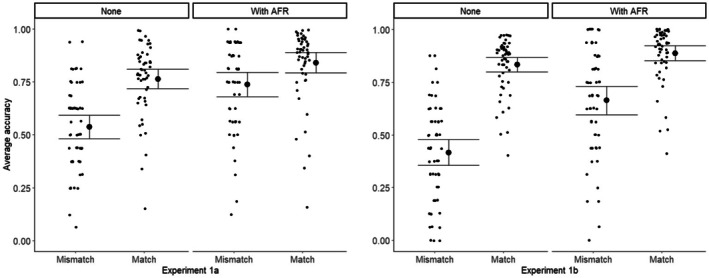
Accuracy (proportion correct) for match (mated) and mismatch (non‐mated) trials, unaided and with the AFR system's guidance for Experiments 1a and 1b. Error bars are 95% CI.

**FIGURE 2:**
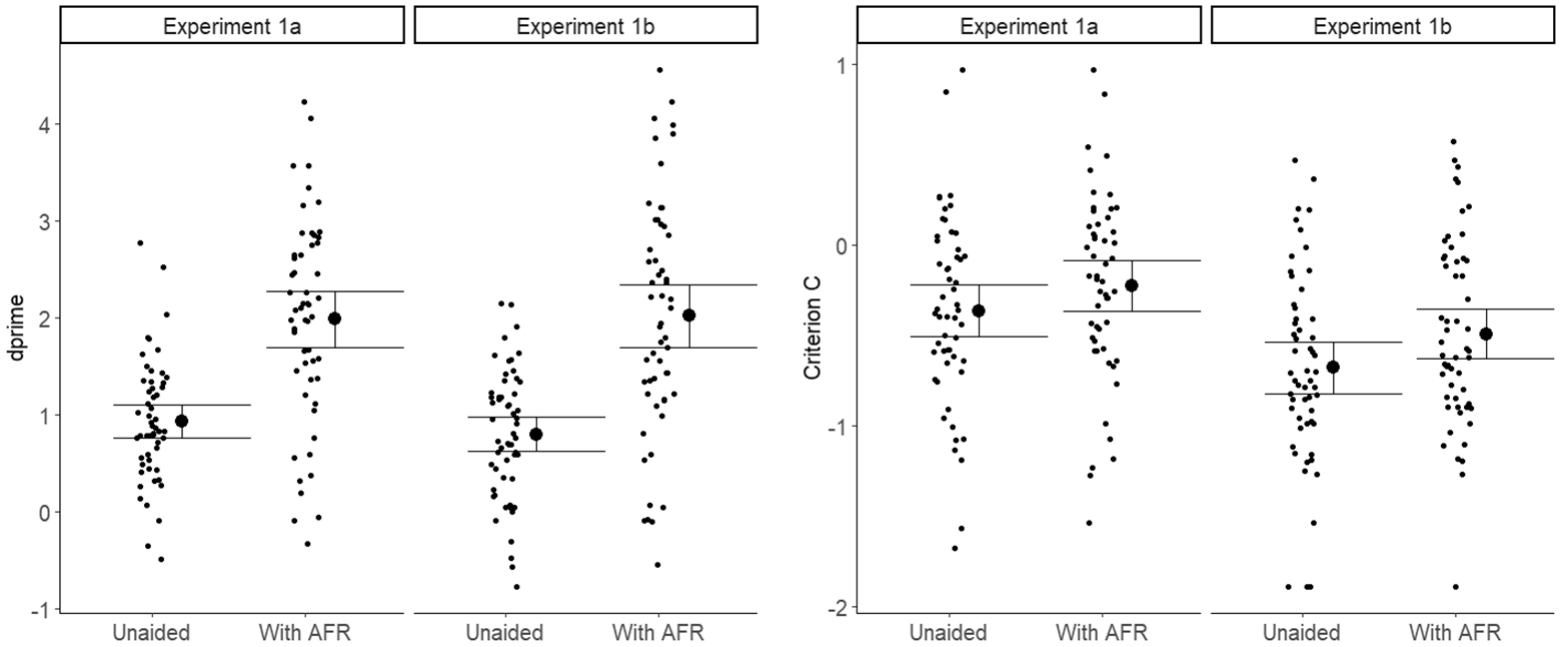
Sensitivity d’ and Criterion C in each condition for Experiments 1a and 1b. Error bars are 95% CI.

Figure 1 shows the accuracy in each condition for Experiments 1a and 1b. It is apparent that accuracy for match pairs is consistently better than for mismatches and that the computer assistance improves performance. A signal detection analysis, shown in Figure 2 and separating sensitivity from bias, makes the comparison between the two experiments clearer.

Sensitivity is almost identical between the two conditions, with the AFR assistance giving a very similar improvement. The difference is in the criterion, which is more negative in Experiment 1b, meaning that participants are biased towards saying match.

A linear mixed effects model for overall accuracy, with participants and face pairs as random factors, is reported in supplementary analysis. A 2 × 2 repeated measure ANOVA, with Aid (unaided, with AFR) as a within‐subjects factor and Experiment as a between‐subjects factor for sensitivity, confirms a large effect of Aid (*F*(1,108) = 162, *p* < .001, η_p_^2^ = .60), but no effect of Experiment (*F*(1,108) = 0.11, *p* = .75, η_p_^2^ = .00) and no interaction (*F*(1,108) = 0.89, *p* = .35, η_p_^2^ = .01). A similar ANOVA for Criterion shows an effect of both Experiment (*F*(1,108) = 9.09, *p* = .003, η_p_^2^ = .08) and Aid (*F*(1,108) = 54.6, *p* < .001, η_p_^2^ = .34) but no interaction (*F*(1,108) = 1.32, *p* = .25, η_p_^2^ = .01).

**FIGURE 3:**
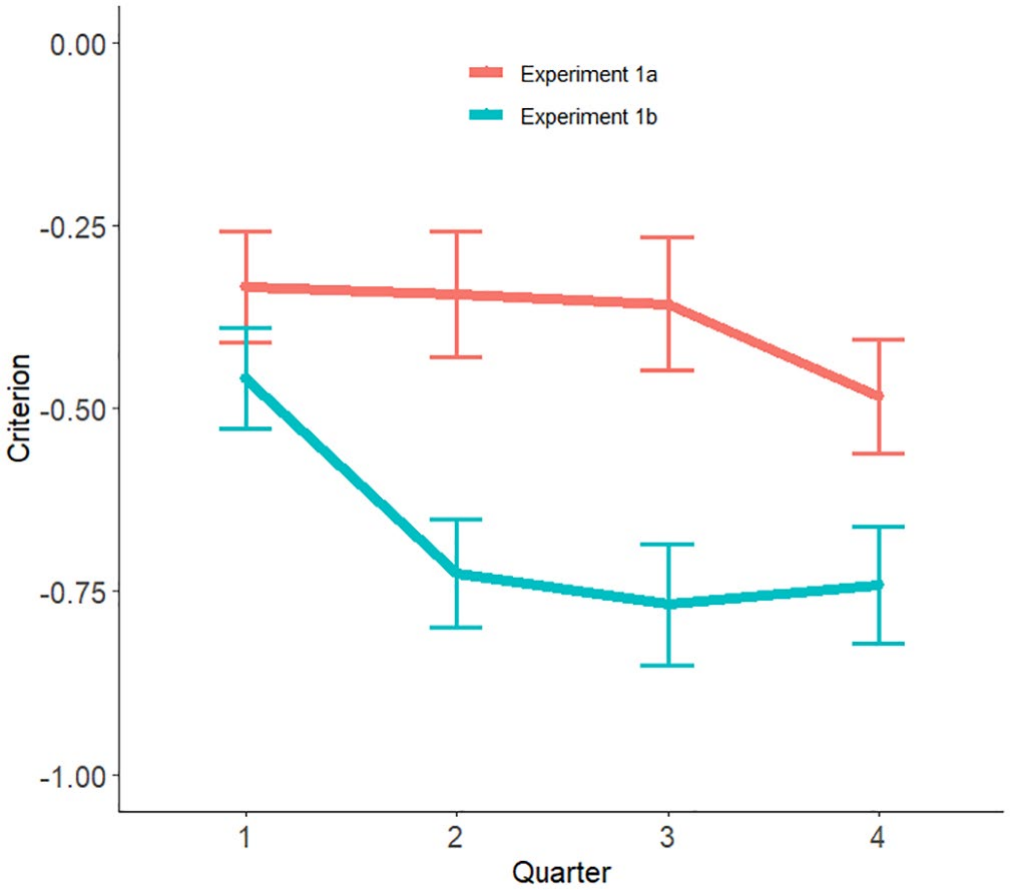
Change in unaided criterion across the four quarters of trials in Experiments 1a and 1b. Error bars are standard errors.

The only difference between the two experiments is the absence of the computer similarity scores in Experiment 1b, leaving only the binary match/mismatch decision. This affects the bias of the participants. Because of the 9:1 ratio of matches to mismatches, participants in both experiments will encounter repeated ‘computer says match’ trials and would be expected to alter their response bias to say match more often (Lynn & Barrett, 2014). In Experiment 1a, the computer decision is moderated by similarity information and it appears our participants are able to interpret that to conclude that while the computer says match, it may not be very certain, and they shift their bias less. How fast the bias changes in Experiment 1b is illustrated in Figure 3, which shows the average criterion when unaided, calculated separately for successive quarters (40/160) of the trials. Since the order of trials was completely randomized, the number of mismatch trials in any set of 40 varied, resulting in some large individual fluctuations in computed biases. However, the average trend is clear: the criterion drops rapidly in Experiment 1b and then stays level, compared to a much more gradual shift in Experiment 1a.

The results so far indicate that the presence of computer similarity information changes participants' bias but has no effect on their overall accuracy. However, there are more subtle effects that are revealed by looking at how the AFR information affected the confidence rating in each experiment. We pre‐registered a ‘change in confidence’ score (CIC), computed as follows:

1. If rating without AFR = rating with AFR: CIC = 0.
2. If rating without AFR < rating with AFR: CIC = (rating with − rating without)/(6 − without).
3. If rating without AFR > rating with AFR: CIC = (rating with − rating without)/(without − 1).

This captures the notion that a given change in confidence is harder to achieve as the top or bottom of the range is approached. It divides the change observed by the maximum possible change. A table and figure illustrating the algorithm are in supplementary analyses.

**FIGURE 4:**
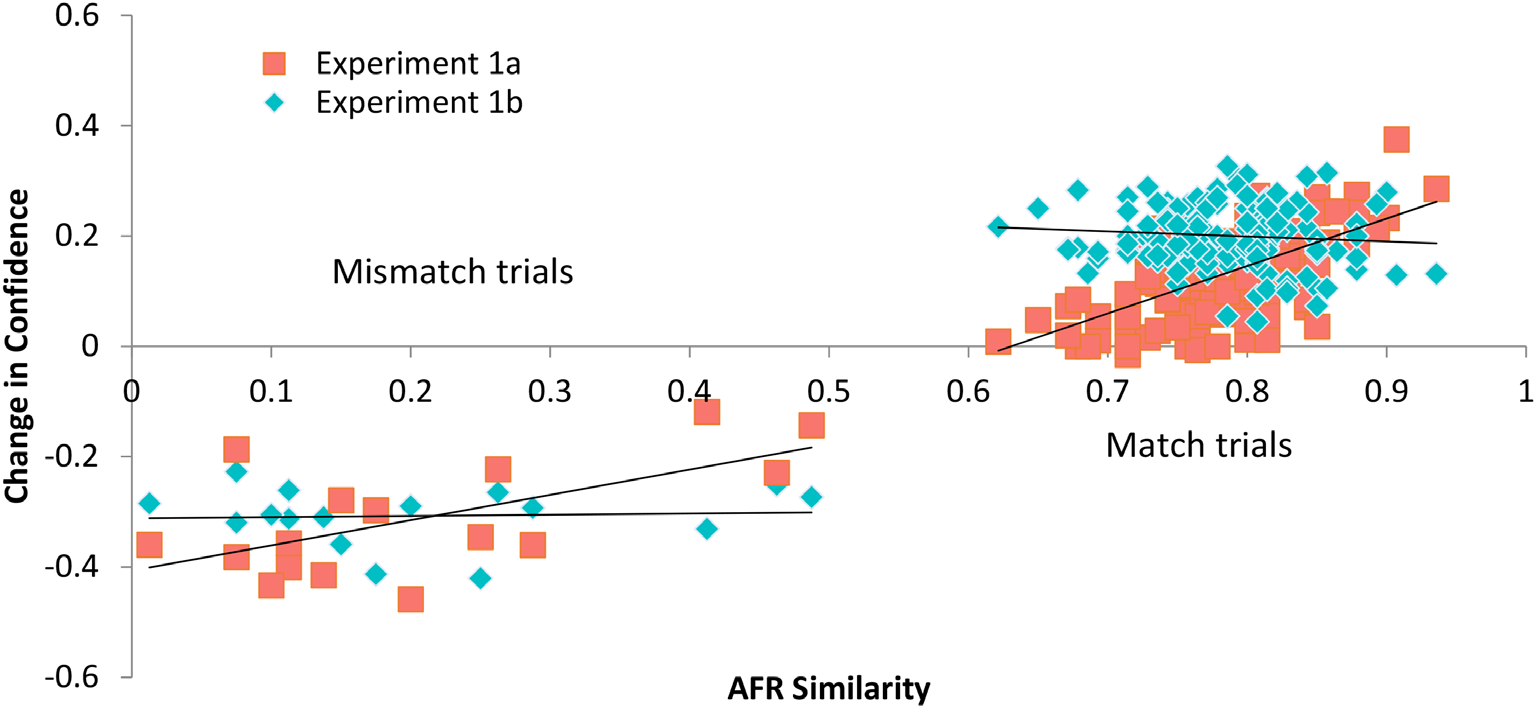
The change in confidence when given the AFR result plotted against the AFR similarity score for each face pair in Experiments 1a and 1b.

Figure 4 shows the change in confidence score plotted against the AFR similarity scores, as given to participants in Experiment 1a. As expected, the changes for match trials are mostly positive, towards a response of 6, which would be a ‘certain match’, while the changes for mismatch trials are negative, in the direction of 1, which would be a ‘certain mismatch’. In Experiment 1a, both regression lines are positive. For match pairs, *r*(142) = .68, *p* < .001 and for mismatch pairs, *r*(14) = .63, *p* = .009. Match pairs that have high AFR similarity, and mismatch pairs that have low AFR similarity, change in confidence in the appropriate direction. Match and mismatch pairs that are near the AFR threshold change in confidence much less. Participants in Experiment 1a use the AFR similarity score to modulate their change in response. By contrast, the regression lines for Experiment 1b are flat. For match pairs, *r(*142) = −.11, *p* = .17 and for mismatch pairs, *r*(14) = .06, *p* = .83. With only a binary response from the AFR in Experiment 1b, participants change their confidence to all pairs equivalently. They are, therefore, relatively overconfident on pairs where the AFR system is least certain. This seems undesirable in a real‐life setting.

Also apparent from Figure 4 is the performance of the AFR system on these face pairs. Although some of the mismatches are close to the boundary of 0.5, there is a clear separation between match and mismatch pairs. The system is 100% accurate on this set.

### Participants' self‐ratings

Experiment 1b contained some additional questions for participants: they were asked how accurate they thought the AFR system was and how accurate they thought they had been, without computer assistance. They were also asked 10 questions about attitudes to AI use, which were combined into a single Trust in AI score by addition. Cronbach's alpha for this scale was 0.84. In the expectation that trust in AI would correlate with how much participants followed the AFR system, we computed an ‘Adherence to AI’ score as (Accuracy with AFR − accuracy unaided)/(1 − Accuracy unaided). Similar to the change in confidence score, this computes a change in accuracy, controlled for the maximum possible increase.

Table 1 shows correlations between these variables. The trust in AI measure did not correlate with any other variable. Participant's estimates of their own and the AFR system's accuracy, and their actual accuracy with and without AFR assistance, all correlate at around *r* = .35. That Adherence to AFR correlates strongly with ‘Accuracy with the AFR’ is unsurprising as the one is calculated from the other. That Adherence also correlates with unaided performance is more interesting, since numerically, a low Unaided score would lead to a high Adherence score. The positive correlation implies that participants who perform better unaided also make better use of the AFR's advice; they improve their scores by a bigger proportion of what is possible than those who do less well.

**TABLE 1:** Pearson correlations between participants' trust in AI, their estimates of their own and of the AFR accuracy and their actual accuracy without and with the AFR result.

	Trust in AI	Self	AFR	Unaided	With AFR
Self accuracy estimate	.01				
AFR accuracy estimate	.08	.35*			
Accuracy unaided	−.13	.34*	.33		
Accuracy with AFR	−.08	.32	.38*	.86*	
Adherence to AFR	−.04	.17	.22	.37*	.73*

* *p* < .01.

#### Experiment 2

The aim of Experiment 2 was twofold. First, in Experiments 1a and 1b, participants' performance improved, but was not perfect, despite the opportunity to be so. This lack of following the AFR aid was unrelated to participants' evaluation of the AFR accuracy or their attitudes towards AFR. It is thus possible that what people and AFR ‘perceive’ as similar differ (see also Hancock et al., 2020; c.f. Ritchie et al., 2023). This ‘misalignment’ can be the source of overruling AFR decision and making mistakes. To test this, we asked a (separate) group of participants to rate the similarity of face pairs used in Experiments 1a and 1b. Second, we wanted to assess human performance on our set when uninfluenced by the AFR scores.

Participants in this study were not told anything about an AFR system. They were presented with the complete set of pairs and asked to rate each pair in turn for similarity on a 100‐point slider scale. They were then presented with the whole set again and asked to make a match decision for each pair, using the same 1–6 scale as in Experiments 1a and 1b.

### Participants

This was a correlational study; G‐Power indicated a sample size of 111 to detect *r* = .3. Our original sample was 134; 17 were excluded for failing attention checks and 6 for taking more than an hour (average time was 35 min), leaving 111, (46 male, 65 female) average age 39, SD 11.8.

### Human and AFR system's similarity

Participants first rated the similarity of each pair of faces on a scale from 0 to 100 and then, in a second block, gave a match rating on the same 1–6 scale used in the first two experiments. Table 2 shows the correlations between the unaided ratings in each experiment and with the AFR similarity score. As expected, the correlations between the ratings are extremely high. The leading diagonal shows the split‐half correlations within each of the human measures. It may be noted that the split‐half correlations for Experiments 1a and 1b are smaller than the correlation between them. This is an expected result of halving the number of participants to do the split.

**TABLE 2:** Correlations between unaided match ratings from Experiments 1a and 1b, the rating and similarity scores from Experiment 2 and the AFR similarity scores, separately for mismatch and match trials. Main numbers are Pearson correlation, and figures in brackets for AFR Similarity are Spearman. Leading diagonal (shaded) are split‐half correlations. All are highly significant except those for mismatch AFR Similarity.

	Expt1a rating	Expt1b rating	Expt2 rating	Expt2 similarity
Mismatches, *n* = 16				
Expt1a rating	.83			
Expt1b rating	.88	.84		
Expt2 rating	.92	.93	.92	
Expt2 similarity	.93	.92	.92	.93
AFR similarity	.40 (.33)	.27 (.24)	.27 (.24)	.43 (.30)
Matches, *n* = 144				
Expt1a rating	.74			
Expt1b rating	.89	.75		
Expt2 rating	.86	.89	.88	
Expt2 similarity	.92	.91	.89	.91
AFR similarity	.49 (.49)	.50 (.49)	.47 (.49)	.54 (.52)

Correlations with AFR similarity are around 0.5 for match pairs. They are numerically smaller for mismatch pairs and non‐significant but with only 16 mismatch pairs it is not safe to infer much from this. Table 2 also reports Spearman rank correlations for the AFR Similarity; the values for the match trials are very similar to the Pearson values, implying that the observed relationships are linear. Scatter plots for key comparisons are in Data S1.

### Effects on accuracy

**FIGURE 5:**
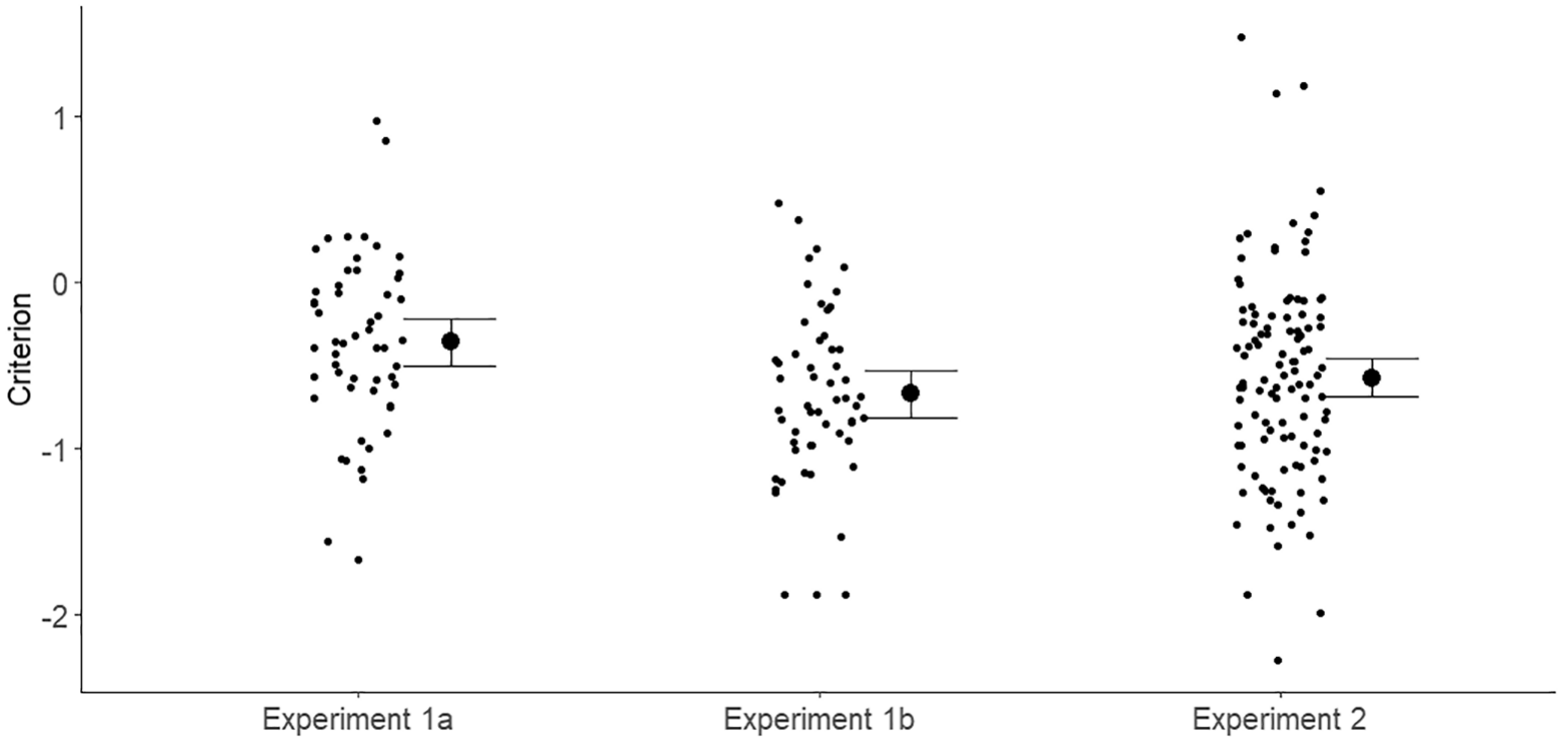
Comparison of criterion between Experiments 1a and 1b, unaided, and Experiment 2. Error bars are 95% CI.

An ANOVA showed that the average d’ scores did not differ between Experiments 1a and 1b when unaided and Experiment 2, *F*(108) = 1.16, *p* = .28, *η*_*p*_^*2*^ = .011. However, Criterion did differ, *F*(108) = 9.95, *p* = .002, *η*_*p*_^*2*^ = .084. Paired tests showed that Criterion was significantly higher (less negative) in Experiment 1a than in Experiment 2, *t*(124.1) = 2.34, *p* = .01, *d* = 0.36, while it was not significantly higher in Experiment2 than in Experiment 1b, *t*(128.2) = 1.08, *p* = .86, *d* = 0.17. Figure 5 shows the average and individual participant Criterion scores for each experiment. Compared with Experiment 2, where there is no computer feedback, and Experiment 1b, where only binary feedback is given from the AFR, the graded feedback in Experiment 1a produces an average Criterion that, while still negative, is closest to zero. The graded AFR scores thus help participants to reduce the bias towards saying match caused by the preponderance of match pairs and, importantly, reduces the chance of missing the critical mismatch items.

## DISCUSSION

Our study set out to examine how people utilize the aid of a highly accurate AFR system when making decisions about two images being of the same person or two different people. To our knowledge, this is the first study to (together) (1) use a face set with naturally occurring ethnicities (here: tracking the London census), (2) present participants with true responses of a highly accurate AFR system and (3) use low mismatch prevalence (10%) more resembling the true fraudulent ID rate than typical matching sets with 50% mismatch prevalence (Burton et al., 2010; Fysh & Bindemann, 2018b). In Experiment 1a, we presented the AFR decision as a similarity score scaled from 0 to 1 and informed participants that the AFR threshold for a match between two images is 0.5. In Experiment 1b, we presented participants with a binary AFR decision (match or mismatch). We found that participants' accuracy improved with the help of AFR in both experiments, but no participants achieved 100% accuracy which would have been possible had they followed the error‐free AFR. The improvements in both experiments were very similar but moderate, as people frequently overruled the AFR. Crucially, we found that in Experiment 1b, where people were given only binary AFR decisions, participants' criterion shifted towards declaring a match response, leading to more errors on the mismatched trials. In Experiment 1b, we also asked participants how accurate they thought they were, how accurate they thought the AFR system was and administered a short questionnaire about their trust in AFR. Participants’ estimate of their unaided accuracy was weakly correlated with their actual performance, the perceived AFR system's accuracy was moderately correlated with aided performance, and there was no relationship between trust in our AFR system and participants' aided performance or the amount by which they improved when aided.

In Experiment 2, we asked a separate group of participants to rate the perceived similarity of the faces (block 1) and their match confidence ratings (block 2) of the same pairs. We found that participants' perceived similarity correlated very strongly with their later confidence responses on the same pairs. Their perceived similarity was also strongly (*r* ~ .5) correlated with the AFR system's similarity scores for match pairs. The correlation for mismatch pairs was weaker, though based on only 16 such pairs in the set. This pattern is, however, consistent with previous findings (e.g. Hancock et al., 2020) that show stronger correlations between humans and AFR for match than mismatch pairs. This is an important finding for researchers (and practitioners) thinking of constructing lineups and matching face sets using AFR instead of human ratings – using AFR to select foils can potentially lead to unfair lineups and negative consequences for the reliability of eyewitness testimony.

### Aided accuracy

Results of Experiments 1a and 1b show that although the confidence and signal detection measures (d prime) are comparable between the two methods of presenting AFR system's decisions (continuous vs. binary), there is a clear criterion shift towards saying ‘match’ when participants get the AFR system's final decision. While in Experiment 1a participants were cautious in following the AFR system when its similarity rating was close to threshold (i.e. more uncertain), in Experiment 1b, they had no means to discern the AFR system's certainty and thus a high prevalence of match responses biased participants towards saying ‘match’.

This bias towards declaring matches was at the cost of accuracy on mismatched pairs. In national security settings, this is a particularly costly mistake, leading to letting a person carrying a fraudulent ID document in or out of the country. One such prominent case was Shamima Begum leaving United Kingdom via Heathrow Airport in 2015 using her older sister's passport (Syria Girls, 2015). We did not observe this strong criterion shift when people were given a continuous similarity score in Experiment 1a suggesting that in applied settings it would be advisable to present human decision‐makers with a similarity, non‐binary, score. We adjusted the raw similarity score to give a range from 0 to 1 and a threshold of 0.5, thinking this would be easier to understand. This assumption might usefully be tested in future work.

The strong criterion shift in Experiment 1b was apparent even in the first quarter of experimental trials and had stabilized about half‐way through the study, remaining low at the cost of mismatch detection. In Experiment 1a, the criterion dropped somewhat after the third quarter of trials, presumably when participants determined that most AFR system's values point towards matched pairs. From the perspective of signal detection theory (SDT), the liberal criterion (tendency towards declaring match) is unsurprising. The utility‐based approach to SDT posits that performance is affected by proportion of matched trials (base rates), the cost/benefit of decisions (payoffs) and perceptual discriminability of the stimuli (in face perception, this can of course depend both on the set of images and individual face processing ability) and that people tend to maximize utility rather than accuracy (Lynn & Barrett, 2014). We did not manipulate the cost/benefit of decisions in this experiment – there was no reward for hits and correct rejections or negative cost associated with misses and false alarms. However, the prevalence of matches and evident medium discriminability of the set prompted participants to respond match more often. It is possible that in national security settings where officials are aware of the cost of missing a fraudulent ID, these biases would be smaller. While Stabile et al. (2024) tried to manipulate these parameters in a laboratory study (without any AFR input), the benefits of virtual tickets or even cash incentive may not be sufficient to mimic national security scenarios where these decisions have real consequences. A study with Police Officers and Border Force staff would elucidate this. In the absence of such data, our study provides first direct evidence that an AFR system's similarity score is beneficial in minimizing misses (declaring a mismatched pair a match) in comparison with a binary AFR system's score in low‐prevalence mismatch environments akin to real‐world scenarios.

### Similarity

Although the computer scores improved the performance in both experiments, the accuracy was not 100%, as would be the case had participants followed the AFR system completely. We deliberately did not include any mistakes and simply informed our participants that the AFR system was extremely accurate in tests, which was a new approach. One reason for the limited improvement could be the discrepancy between what people and AFR find as similar. In Experiment 2, we thus asked different group of participants to rate the similarity of the same pairs on a scale from 0 to 100 in Block 1 and then decide whether the pairs were same or different using the 1–6 rating scale. Although participants' similarity ratings are nearly perfectly correlated with the later confidence response (*r* > .9), the correlations between the AFR system's and human similarity ratings were only medium (.54 and .43 for matches and mismatches, respectively). This moderate correlation is in line with the findings of Stantic et al. (2022; Studies 1, 2 and 3) on which we based our similarity scale (0–100). Stantic and colleagues constructed their Oxford matching test using stimuli selected by a state of the art algorithm, but while pointing to several advantages of this approach (objective measure not depending on one group of participants, ability to construct parallel stimuli sets), they also acknowledge that similarity judgements differ between humans and AFR and that different algorithms base their similarity judgements on different metrics leading to discrepant scores on some face pairs. A recent study by Ritchie et al. (2023) reported a relatively strong correlation between AFR and human raters, but this was across both match and mismatch trials, rather than separately as here.

What might be the effect of human raters and the AFR system having different notions of similarity? Suppose the AFR says match for a pair that look obviously different, such as being apparently a different sex (Hancock et al., 2020). A user might conclude the system is of little use and ignore its advice. However, given that both humans and the AFR will make mistakes, the optimal situation might be for them to make different mistakes and for the human to override ‘obvious’ AFR errors but to defer to it when they are uncertain. Phillips et al. (2018) argue that it is indeed best for experts, human or AFR, to make different errors when their outputs will be fused, that is, combined optimally to give a final answer. In our experiments, data are not fused; humans are deciding based on their own judgement, with input from the AFR. Experiment 3 of Carragher and Hancock (2023) tested the effects of the AFR making errors either on pairs that humans found easy or hard. Surprisingly, there was no difference in the aided performance. Nevertheless, as AFR continue to improve and their use extends to more marginal cases, it seems important for users to have a good understanding of the ways a particular system may fail. For example, identical twins continue to be a challenging problem for both humans and AFR.

### Self‐reported ability and trust in AFR

Participants in Experiment 1b were asked to estimate their own unaided accuracy on the task, how accurate the AFR was and a series of questions to assess their trust in AFR which we adopted from Ezzeddine et al. (2023). We found a weak correlation between self‐reported accuracy and actual unaided accuracy (*r* = .34). This is in line with previous work on self‐reported face processing ability (Bobak et al., 2019; Kramer, 2021; Palermo et al., 2017) and in line with people's cross‐domain ability estimations and objective performance (Zell & Krizan, 2014). Recently, Kramer and Tree (2023) suggested that one of the reasons why self‐reported ability and actual ability correlations are weak may be due to different questions tapping into various domains. However, our question was phrased to estimate performance on the test participants had just taken suggesting that even this very focussed estimation is poor. Participants' perceptions of AFR accuracy were also only weakly correlated with their aided performance (*r* = .38) reflecting the frequent overruling of AFR and ultimately, making mistakes in our test. We hoped a relationship between (lack of) trust in AFR and actual aided accuracy may explain this result, but the self‐reported trust was not related to participants' aided performance. As attitudes towards AFR differ greatly between countries and types of use (Ritchie et al., 2021), future studies may wish to examine the interplay between the type of use, individual attitudes and human–computer interaction like that required by our studies.

### Ecologically valid approaches to AFR‐aided face matching

Our study adopted an unorthodox, more ecologically valid approach to studying how AFR aids face processing. First, we devised a face set that, akin to Papesh and Goldinger (2014), included naturally occurring ethnicities from the Greater London area (based on the 2021 census). Second, we used a low‐prevalence, 10%, mismatch rate in our set that departs from the traditional 50% rate used by most studies (c.f. Fysh & Bindemann, 2018a; Papesh & Goldinger, 2014). Third, we presented participants with the true AFR decisions (both continuous and binary) and informed them that our system was over 99% accurate in tests with large similar data sets. Finally, we recruited a diverse sample of participants from the London area. Although this approach departs significantly from typical restricting of ethnicity in face sets and samples, we think our approach is better representing psychology of homo sapiens, rather than WEIRD samples (Henrich et al., 2010). For instance, travellers to the biggest UK airport, London Heathrow, are from all over the world. Heathrow employees are likely to come from Greater London area and be ethnically diverse thus our study approximates better the environment of passport control on this major travel hub.

In their target article, Ramon et al. (2019) advocated for laboratory tasks closely resembling real world scenarios and questioned the utility of most used tests in predicting real‐life performance. This translational approach is now widely adopted in Ramon's work with various police forces (e.g. Mayer & Ramon, 2023). We think our matching task is a step closer to mimicking the applied settings in line with these recommendations and would be generalizable to people undergoing identity verification in large cities.

## CONCLUSIONS

*A*utomated facial recognition (AFR) systems are becoming increasingly common in society, but require a human in the loop providing oversight (Carragher & Hancock, 2023; Fysh & Bindemann, 2018a; White et al., 2015). Our study was first to use true outcomes of a state‐of‐the art AFR system in a diverse set of faces with a low‐prevalence mismatch rate and recruiting diverse participants from London area. Despite accurate information given to participants about the AFR system being highly accurate, participants regularly overruled the AFR system, leading to small improvements in performance. Concerningly, when given binary AFR decision (match, mismatch) participants rapidly adopted a liberal criterion and shifted towards responding match at the cost of mismatch trials, a potentially costly mistake in applied settings. We found no evidence for the aided accuracy's relation to trust in AFR and little relationship between participants' estimations of the AFR system's accuracy and their aided performance. This bias was smaller and slower to emerge when participants were given the AFR similarity score. Crucially, participants given only the binary outcome were relatively over‐confident on face pairs that were near the AFR's threshold. Participants given the similarity information made use of it. For these reasons, we recommend that users should always be given information about the AFR similarity score. We suggest that it will also be helpful to educate users about the nature of errors made by the AFR systems and that how best to do this should be a focus of future research.

## AUTHOR CONTRIBUTIONS

**Melina Mueller:** Conceptualization; investigation; methodology. **Peter J. B. Hancock:** Conceptualization; writing – original draft; methodology; visualization; writing – review and editing; formal analysis; data curation. **Emily K. Cunningham:** Investigation. **Roger J. Watt:** Conceptualization; methodology. **Daniel Carragher:** Writing – original draft. **Anna K. Bobak:** Conceptualization; investigation; funding acquisition; writing – original draft; methodology; writing – review and editing; project administration; supervision; resources.

## CONFLICT OF INTEREST STATEMENT

The authors have no conflict of interest to report.

## Supporting information


Data S1:

## Data Availability

The data that support the findings of this study are openly available in Open Science Framework at https://osf.io/mxnka/?view_only=f1aee44a79a34cd78b52972993821da0.
