# Supplementary material for: Automated face recognition assists with low‐prevalence face identity mismatches but can bias users
Source: Br J Psychol. 2024 Nov 15;117(2):567–84. doi: 10.1111/bjop.12745 (PMC13051006; doi:10.1111/bjop.12745)

**Automated face recognition assistance with low-prevalence face identity mismatches**

**Supplementary materials**

**Participants.** We asked our participants to self-describe their gender identity and ethnicity.

**Experiment 1**: 54 Participants, average age 39, Sd 11.8

| Gender | **female** | **M** | **Male** | **woman** | **women** |
| --- | --- | --- | --- | --- | --- |
| n | 26 | 1 | 25 | 1 | 1 |

| Ethnicity | **Asian** | **black** | **Black-British** | **British** | **British Indian** | **Caribbean** |
| --- | --- | --- | --- | --- | --- | --- |
| n | 6 | 2 | 1 | 5 | 3 | 1 |
| Ethnicity | **Caucasian** | **Chinese** | **Indian** | **irish** | **Kurdish** | **Mixed** |
| n | 2 | 1 | 2 | 1 | 1 | 1 |
| Ethnicity | **Mixed Asian and Caucasian** | **Mixed Race** | **NA** | **prefer not to say** | **Russian** | **Somali** |
| n | 1 | 1 | 1 | 1 | 1 | 1 |
| Ethnicity | **South Asian** | **UK** | **Vietnamese** | **White** | **White British** | **White English** |
| n | 1 | 1 | 1 | 14 | 4 | 1 |

**Experiment 2**: 56 Participants, average age 40, sd 11.5

| Gender | **Bloke** | **F** | **Female** | **Male** | **non-binary** | **Sex is female / no gender identity** | **She/They** | **woman** |
| --- | --- | --- | --- | --- | --- | --- | --- | --- |
| n | 1 | 1 | 30 | 20 | 1 | 1 | 1 | 1 |

| Ethnicity | **Arab** | **Asian** | **Asian or Asian British - Indian** | **black** | **Black African** | **Black British** |
| --- | --- | --- | --- | --- | --- | --- |
| n | 2 | 4 | 1 | 1 | 1 | 1 |
| Ethnicity | **British** | **British Asian** | **British black** | **Caucasian** | **Chinese** | **Eastern European** |
| n | 6 | 1 | 1 | 2 | 2 | 1 |
| Ethnicity | **hispanic** | **Mixed** | **Mixed race - Black/White** | **Mixed Race (White & Asian)** | **Mixed: White British / Black Caribbean** | **Pakistani** |
| n | 1 | 1 | 1 | 1 | 1 | 1 |
| Ethnicity | **White** | **White british** | **White/caucasian** |  |  |  |
| n | 18 | 7 | 2 |  |  |  |

**Experiment 3** 111 Participants, average age 39, sd 11.8

| Gender | **Cis female** | **femail** | **female** | **Female / woman** | **I don't believe in gender identity, my sex is female** |
| --- | --- | --- | --- | --- | --- |
| n | **1** | **1** | **58** | **1** | **1** |
| Gender | **m** | **Male** | **Man** | **Sex: Male** | **woman** |
| n | **2** | **42** | **1** | **1** | **3** |

| Ethnicity | African | asian | Bangladeshi | black | Black African | Black british | Black Caribbean |
| --- | --- | --- | --- | --- | --- | --- | --- |
| n | 1 | 6 | 1 | 3 | 2 | 3 | 1 |
| Ethnicity | brazilian | british | British African | British Asian | British Indian | caucasian | caucasian white |
| n | 1 | 14 | 1 | 2 | 1 | 2 | 3 |
| Ethnicity | Chinese | East Asian | Eastern European | english | european | hungarian | indian |
| n | 3 | 1 | 1 | 2 | 1 | 1 | 3 |
| Ethnicity | Irish | jamaican | Latino | litthuanian | mixed | mixed european and asian | Mixed white |
| n | 1 | 1 | 1 | 1 | 8 | 1 | 1 |
| Ethnicity | Multi-ethnic | Persian | White | White - Other | White Asian (British Nationality) | White British | white european |
| n | 1 | 1 | 24 | 1 | 1 | 12 | 2 |
| Ethnicity | white jewish | White, British |  |  |  |  |  |
| n | 1 | 1 |  |  |  |  |  |

**Trust in AI.** Participants in Experiment 2 were asked 10 questions about Trust in AI, adapted from Ezzeddine 2023, with two extra attention check questions

| Q1 | I would falsify my personal information online to protect my privacy from AI systems. | Negative |
| --- | --- | --- |
| Q2 | I ask my family and friends to never post pictures of me on their social media to avoid AI systems collecting and using them. | Negative |
| Q3 | I am happy for AI systems to monitor and use my personal information to safeguard others from terrorism/cybercrime. | Positive |
| Q4 | I avoid public spaces such as street festivals or airports to stop AI systems from capturing my face and movements. | Negative |
| A1 | Select "Totally agree" for this statement. |  |
| Q5 | I am happy for national security agencies to use AI as they see fit for public safety. | Positive |
| Q6 | I am happy for the AI systems to use my personal information (images and data) to learn how to identify bad actors (terrorists, cybercriminals). | Positive |
| Q7 | I trust AI systems to identify people accurately. | Positive |
| A2 | Answer with "Somewhat disagree". |  |
| Q8 | AI systems are helpful for monitoring crowds and flagging up suspicious behaviour. | Positive |
| Q9 | I would be happy if police facial recognition cameras appeared on my street. | Positive |
| Q10 | I think AI can help national security agencies (Border Force, Police) to be more accurate and efficient. | Positive |

**Area Under the Curve** results for Experiments 1 and 2

**Linear mixed effects models.** Procedure in each case was to start with full model and remove terms from random effects until the model converged, using lme4 and lmertest in R 4.2.2

**Experiment 1 accuracy** Effect of match/mismatch, AFRS aid and interaction between them.

glmer(correct ~ faceType*infoType + (1 + faceType + infoType || subject) + (1 | trialno), data = expt1_data,family = "binomial")

Estimate Std. Error z value Pr(>|z|)

faceType 1.1503 0.3325 3.459 0.000542 ***

infoType 0.9529 0.1069 8.915 < 2e-16 ***

faceType:infoType -0.4280 0.1307 -3.275 0.001057 **

**Experiment 1 rating, matches** Effect of AFRS aid

lmer(rating ~ infoType + (1 + infoType || subject) + (1 | trialno), data = expt1_matches, REML=FALSE)

Estimate Std. Error df t value Pr(>|t|)

infoType 0.21271 0.03000 53.99986 7.091 2.93e-09 ***

**Experiment 1 rating, mismatches** Effect of AFRS aid

lmer(rating ~ infoType + (1 + infoType || subject) + (1 | trialno), data = expt1_mismatches, REML=FALSE)

Estimate Std. Error df t value Pr(>|t|)

infoType -0.69213 0.06894 53.99977 -10.04 5.94e-14 ***

**Experiment 2 Accuracy** Effect of match/mismatch, AFRS aid and interaction between them.

glmer(correct ~ faceType*infoType + (1 + faceType+infoType || subject) + (1 | trialno), data = expt2_data,family = "binomial")

Estimate Std. Error z value Pr(>|z|)

faceType 2.2897 0.3455 6.628 3.40e-11 ***

infoType 1.1001 0.1191 9.234 < 2e-16 ***

faceType:infoType -0.5948 0.1334 -4.459 8.24e-06 ***

**Experiment 2 rating, matches** Effect of AFRS aid

lmer(rating ~ infoType + (1 + infoType || subject) + (1 | trialno), data = expt2_matches, REML=FALSE)

Estimate Std. Error df t value Pr(>|t|)

infoType 0.24120 0.03835 56.00262 6.289 5.12e-08 ***

**Experiment 2 rating, mismatches** Effect of AFRS aid

lmer(rating ~ infoType + (1 + infoType || subject) + (1 | trialno), data = expt2_mismatches, REML=FALSE)

Estimate Std. Error df t value Pr(>|t|)

infoType -0.86607 0.09423 56.00059 -9.191 8.88e-13 ***

**Experiment 1 and 2 compared, accuracy.** Effect of AFRS aid, interaction between match type and Experiment only

glmer(correct ~ faceType*infoType * Experiment + (1 + infoType || subject) + (1 | trialno), data = combined_data, family = "binomial")

Estimate Std. Error z value Pr(>|z|)

faceType 0.064824 0.242789 0.267 0.789470

infoType 0.843459 0.235155 3.587 0.000335 ***

Experiment -0.005583 0.175245 -0.032 0.974587

faceType:infoType -0.270181 0.276749 -0.976 0.328933

faceType:Experiment 1.001917 0.087223 11.487 < 2e-16 ***

infoType:Experiment 0.071658 0.147807 0.485 0.627811

faceType:infoType:Experiment -0.157578 0.173743 -0.907 0.364428

**Experiment 1 and 2 rating compared, matches.** Effect of AFRS aid and experiment, no interaction

lmer(rating ~ infoType * Experiment + (1 + infoType || subject) + (1 | trialno), data = combined_matches, REML=FALSE)

Estimate Std. Error df t value Pr(>|t|)

infoType 0.18422 0.07776 110.01084 2.369 0.019583 *

Experiment 0.44892 0.12732 109.95245 3.526 0.000617 ***

infoType:Experiment 0.02849 0.04892 110.01084 0.582 0.561473

**Experiment 1 and 2 rating compared, mismatches.** Effect of AFRS aid and experiment, no interaction

lmer(rating ~ infoType * Experiment + (1 + infoType + Experiment || subject) + (1 | trialno), data = combined_mismatches, REML=FALSE)

Estimate Std. Error df t value Pr(>|t|)

infoType -0.5182 0.1867 110.0006 -2.776 0.00648 **

Experiment 0.4588 0.1449 109.2730 3.165 0.00201 **

infoType:Experiment -0.1739 0.1174 110.0006 -1.481 0.14142

**Scatter plots showing the correlations between the ratings in each experiment:**

The diagonal line is the line of equality. Most points lie below it: unaided participants in Experiment 1 gave somewhat lower match ratings on average than in Experiment 3.

Again. the diagonal line is the line of equality. Most points lie above it: unaided participants in Experiment 2 gave somewhat higher match ratings on average than in Experiment 3.

The similarity and match confidence scores given in Experiment 3 correlate very strongly. There is a hint of an upward curve.

This shows the relationship between human similarity scores from Experiment 3 and the raw AFR Similarity scores, prior to the adjustment presented to participants. The original threshold for declaring a match is 0.3.

**Change in Confidence score**

We wished to report an indication of how much influence the AFR information had on participants’ confidence scores. The simplest measure is a difference: rating with AFR - rating without AFR. However, this does not capture the relative size of the change: going from 2 to 3 is one step out of a possible 4 before the maximum of 6, while going from 4 to 5 is one out of 2. So we initially thought just to divide the change by the possible change, so by 4 when going from 2 to 3 and by 2 when going from 4 to 5. The problem arises when the unaided score is a 6, in which case the maximum possible change is zero and a divide-by-zero error occurs. We therefore developed the following algorithm:

1) if rating without AFR = rating with AFR : CIC=0
2) if rating without AFR < rating with AFR: CIC= (rating with - rating without)/(6-without)
3) if rating without AFR > rating with AFR: CIC= (rating with - rating without)/(without-1)

This is bounded at +/-1; example values are shown in this table

|  | Rating with AFR | | | | | |
| --- | --- | --- | --- | --- | --- | --- |
| Rating without | 1 | 2 | 3 | 4 | 5 | 6 |
| 1 | 0.00 | 0.20 | 0.40 | 0.60 | 0.80 | 1.00 |
| 2 | -1.00 | 0.00 | 0.25 | 0.50 | 0.75 | 1.00 |
| 3 | -1.00 | -0.50 | 0.00 | 0.33 | 0.67 | 1.00 |
| 4 | -1.00 | -0.67 | -0.33 | 0.00 | 0.50 | 1.00 |
| 5 | -1.00 | -0.75 | -0.50 | -0.25 | 0.00 | 1.00 |
| 6 | -1.00 | -0.80 | -0.60 | -0.40 | -0.20 | 0.00 |

When comparing average match ratings across participants, the values will be non-integral; this figure visualizes the range of the function.


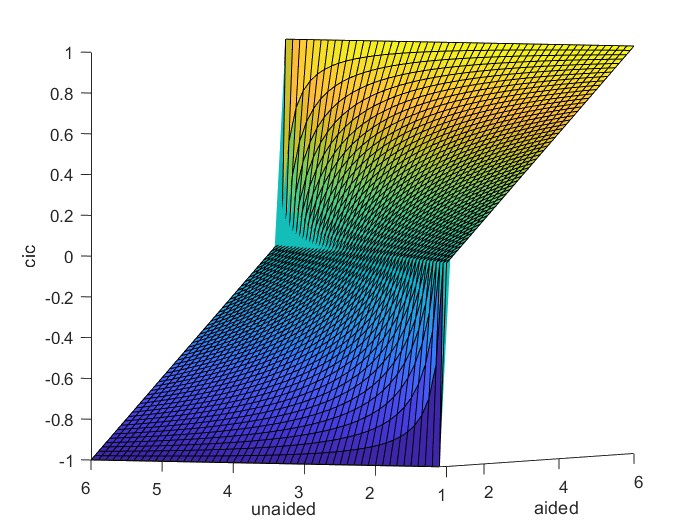

Supplement: Supplementary file 1 — Data S1: [file BJOP-117-567-s001.docx]
